# The Impact of Statin Use and Breast Cancer Recurrence - A Retrospective Study in Singapore

**DOI:** 10.3389/fonc.2022.835320

**Published:** 2022-03-31

**Authors:** Yirong Sim, Cindy Lim, Nitar Phyu, Kiat Tee Benita Tan, Lita Sui Tjien Chew, Chow Yin Wong, Preetha Madhukumar, Wei Sean Yong, Sue Zann Lim, Julie Liana Bte Hamzah, Si Ying Tan, Wen Yee Chay, Fuh Yong Wong, Puay Hoon Tan, Veronique Kiak-Mien Tan

**Affiliations:** ^1^ Department of Breast Surgery, Division of Surgery and Surgical Oncology, National Cancer Centre Singapore, Singapore, Singapore; ^2^ Department of Breast Surgery, Singapore General Hospital, Singapore, Singapore; ^3^ SingHealth Duke-National University of Singapore (NUS) Breast Centre, Singapore, Singapore; ^4^ Clinical Trials and Epidemiological Sciences (CTE), National Cancer Centre Singapore, Singapore, Singapore; ^5^ Department of Cancer Informatics, National Cancer Centre Singapore, Singapore, Singapore; ^6^ Department of General Surgery, Sengkang General Hospital, Singapore, Singapore; ^7^ Department of Pharmacy, National Cancer Center Singapore, Singapore, Singapore; ^8^ Division of Medical Oncology, National Cancer Centre Singapore, Singapore, Singapore; ^9^ Division of Radiation Oncology, National Cancer Centre Singapore, Singapore, Singapore; ^10^ Division of Pathology, Singapore General Hospital, Singapore, Singapore

**Keywords:** statin, HMG-CoA reductase inhibitor, breast cancer, recurrence, Asia

## Abstract

**Introduction:**

Statins, HMG-CoA reductase inhibitors, are commonly used cholesterol-lowering medications which are also increasingly recognized to have anti-cancer properties for various cancers, including breast cancer. Most clinical evidence supports a protective effect of statin on reducing breast cancer recurrence, particularly in hormone-receptor positive breast cancers.This study seeks to study the impact of statin use on breast cancer recurrence in an Asian population.

**Methods:**

This is a retrospective study of patients diagnosed with breast cancer at the National Cancer Centre and Singapore General Hospital from 2005-2015. Statin use was defined as use after surgery. Associations between statin use, breast cancer recurrence and overall survival were estimated using Cox proportional hazards regression with adjustment for age, TNM stage, grade, ER/HER2 status, and co-morbidities. Associations between statin-use and disease-specific survival were estimated using competing risks regression.

**Results:**

A total of 7858 females with breast cancer were studied, 1353(17.2%) were statin users, 6505(82.8%) were non-statin users, with a median follow-up of 8.67 years. Distribution of cancer stage, histology, molecular subtypes and grades were similar in both groups. Estrogen receptor(ER) positive (HR 0.57,95%CI 0.43-0.76,p<0.001) and HER2 negative (HR 0.74,95%CI 0.57-0.96,p=0.026) invasive cancers had a lower risk of recurrence in statin users. Statin users trended towards a long term recurrence-risk reduction (all subtypes,HR 0.48,p=0.002; ER-, HR 0.34,p=0.036; HER2+,HR 0.10,p=0.002). The risk-reduction benefit is not appreciated in statin users with DCIS, possibly due to small recurrence event numbers. Disease-specific survival benefit was seen in statin users with ER+ cancers (adjusted SHR 0.71,95%CI 0.53-0.96,p=0.027), especially ER+ invasive cancers (adjusted SHR 0.72, 95%CI 0.53-0.97,p=0.028), but with no statistically significant benefit in overall survival for statin users (all subtypes).

**Conclusion:**

This is the first known retrospective study on the effect of statin use and breast cancer recurrence in an Asian population. Similar to previous international studies, statin use is associated with a risk reduction in breast cancer recurrence. This is especially beneficial in patients who have ER+ and HER2- invasive breast cancer. Statin use is also associated with a reduced risk of breast cancer recurrence in all subtypes of breast cancer in the long term (>6 years post diagnosis).

## Introduction

Breast cancer is the most commonly occurring cancer in women, and is generally subtyped according to its expression of three receptors - estrogen (ER), progesterone (PR) and HER2 receptors. The majority (67-80%) of breast cancer cells express the estrogen and/or progesterone hormone receptors (ER and PR) (1, 2). With increased screening, improved surgery and adjuvant treatment modalities (i.e. a combination of hormonal, chemo-, targeted and radiation- therapies), the long-term survival rate after the diagnosis of breast cancer is rising steadily. Each breast cancer survivor has a life-long risk (5-41%) of developing a breast cancer recurrence (3–5), and with an increasing number of breast cancer survivors, this translates to an important public health issue. Recurrence can be local, i.e. developing in or near the original site of cancer; regional, i.e. presenting as nodal involvement in the axillary, or supraclavicular anatomic locations; or distant, i.e. metastatic, commonly appearing in the bones, lung, liver, or brain.

Statins are 3-hydroxy-3-methylglutaryl–coenzyme A (HMG-CoA) reductase inhibitors and decrease cholesterol synthesis. They are commonly used as a cholesterol-lowering medication to reduce the risks of heart attacks and strokes. Statins have also been shown to have anti-cancer properties for various cancers, including breast cancer (6–11). Statin users showed a risk reduction in developing breast cancer (12), and breast cancer patients who used cholesterol lowering medicines had more favorable tumour characteristics and improved outcomes compared with nonusers (13). Most clinical evidence supports a protective effect of statins on reducing breast cancer recurrence (14–19).

This benefit appears to be strongest in younger patients (18) and early stage breast cancer (14, 18), suggesting a longitudinal influence of statin therapy (14, 16, 18). Some studies suggest the benefit of statins with hormone receptor positive breast cancers (18, 20), and not with the triple negative breast cancer (TNBC) cohort, i.e. breast cancer cells which do not express the estrogen, progesterone and HER2 receptors (21, 22). There is some suggestion of the lipophilic statins (such as simvastatin) being more effective at reducing breast cancer recurrence than the hydrophilic statins (15, 17, 19). To date, multiple meta-analyses yield conflicting results. Whilst some studies do not show an effect of statins on cancer incidence or survival (23–25), the majority of the meta-analyses support the finding that statins reduce the risk of breast cancer recurrence and breast cancer related mortality (17, 19, 26, 27), particularly during shorter term follow-up (27).

To date, most studies are performed in the Western population, hence there is an importance to perform this study in our local Asian population. The demographics of breast cancer in the East differ from the West, with a peak age of incidence of breast cancer in the East being 10 years younger than the West (28). There are also racial differences in the response to statins, due to genetic determinants and pharmacokinetic differences (29). Therefore, the aim of this study is to retrospectively review and explore the impact of statin use on breast cancer recurrence in the Asian population of Singapore.

## Methods and Materials

**Figure 1:**
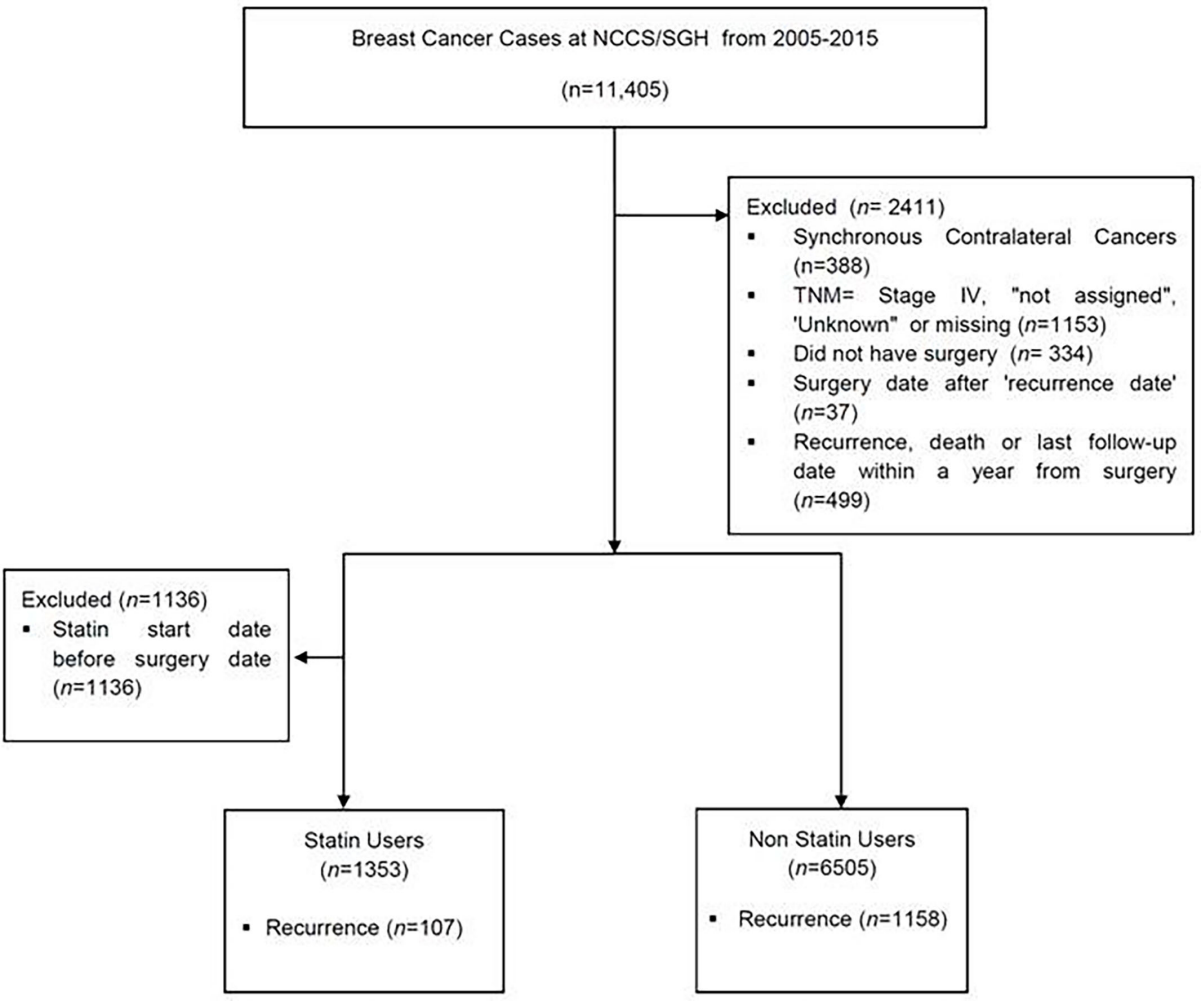
CONSOT Diagram to summarize the number of patients included and excluded in this study. NCCS, National Cancer Centre Singapore; SGH, Singapore General Hospital; TNM, Tumor Nodal Metastasis staging.

This is a retrospective study of patients who were diagnosed with breast cancer at the National Cancer Centre Singapore and Singapore General Hospital from 2005 to 2015, inclusive. The data was extracted from the Joint Breast Cancer Registry (JBCR) and merged with the electronic drug prescriptions platform used by the public health institutions within the SingHealth cluster, which include the primary care physician clinics. This was done with approval by the SingHealth institutional ethics board (CIRB 2019/2419). Breast cancer patients with ductal carcinoma *in situ* (DCIS), Stage I to Stage III invasive cancers were included. Patients whose diagnosis date was before 1 Jan 2005, cancers which were stage IV or unknown/incomplete, and patients who did not undergo surgery were excluded. Patients who had recurrence or died or whose last follow-up date within a year from surgery were excluded (**Figure 1**). For patients who had synchronous bilateral cancers, the following criteria were used to select the side to be included in the analysis in this order: 1) higher TNM stage, 2) higher T stage, 3) more aggressive tumour biology (eg. HER2 positive or TNBC), 4) higher grade, 5) larger tumour size, 6) earlier diagnosis date. If all these 6 criteria were all identical, the left breast was selected by default.

Statin use was defined as use after surgery, as represented by their prescription date. Patients who were on statins pre-surgery were excluded. Patients who developed a recurrence before their start date of statins were counted as statin non-users. The median duration from surgery to start of statins was 22.7 months (IQR 0.36-64.2).

Follow-up started 1 year from breast cancer surgery. Maximum length of follow-up was set to 10 years (or 11 years from surgery). Breast cancer recurrence was defined as any local, regional or distant recurrence, or contralateral breast cancer. Patients who did not have recurrence were censored at death, last follow-up, or when 10 years of follow-up was reached (whichever came first).

### Statistical Methods

Patient and tumour characteristics were tabulated by statin use. Differences in characteristics between statin users and non-users were tested using Pearson’s chi-squared test or Fisher’s exact test (for variables with category frequencies of less than 5). Associations between statin use and breast cancer recurrence and overall survival were estimated using univariable and multivariable Cox proportional hazards regression models. Associations between statin use and disease-specific survival were estimated using univariable and multivariable competing risks regression models as described by Fine and Gray (30). Multivariable models were adjusted for age at diagnosis, TNM stage, histological grade, ER status, HER2 status, aspirin use (pre- and post-diagnosis) and the presence of the following co-morbidities at diagnosis: diabetes, hypertension, cardiac disease, neurological (CNS) disease, hyperlipidemia and renal disease. Statin and aspirin use were fitted as time-varying variables lagged by 1 year, i.e. patients only joined the statin group 1 year after they started on statins. Once started, these drugs were assumed to be taken for life. The proportional hazards assumption was tested using Schoenfeld residuals for Cox regression, and time-varying covariates for competing risks regression. Variables which were significantly non-proportional were modeled using a time-dependent interaction term which allowed the hazard ratio to change after 5 years. The hazard ratio for 6 to 10 years was obtained by multiplying the hazard ratio for 1 to 5 years by this interaction term.

The risk is evaluated according to time from enrolment. For statin users, they are in the nonuser group before statin use initiation and move to the statin group 1 year after statin initiation. The Cox model evaluates hazard rate at each timepoint, which is the risk of failure given the subject has survived up to that timepoint, hence the comparison is between statin users and nonusers who have survived for the same amount of time, and who have been on statins for at least 1 year (for the statin users). All statistical analyses were performed in Stata 16.1 (Statacorp, College Station, Texas). Two-sided p-values less than 0.05 were taken as significant.

## Results

In this study, there were a total of 7858 females with breast cancer were studied, 1353 (17.2%) were statin users, 6505 (82.8%) were non-statin users (**Figure 1**). The majority of the patients were prescribed a lipophilic statin (n=1303, 96.3%), with simvastatin and atorvastatin being the most common statins used. Hydrophilic statins contributed to a marked minority (n=50, 3.7%), with rosuvastatin being the most common hydrophilic statin used (n=47). The demographics and tumor characteristics of the study population is summarized in **Table 1**. The majority of the women in the statin group were postmenopausal. This is expected as the average age in the statin user group was higher, a reflection of statin prescription for cardiac protection in the older population. Nonetheless, the distribution of the cancer stage, histology, tumour molecular subtypes and grade are similar in both groups. As such the use of chemotherapy and hormone therapy are also similar in both groups. In terms of surgery, slightly more women in the statin group chose a mastectomy compared to the non-statin group (65.6 *vs* 55.6%, p<0.001), and hence the uptake of radiotherapy (which is coupled with breast conserving surgery) was lower in statin group (54.4% *vs* 61.5%, p<0.001). The median age in the mastectomy group was also higher (54 years, IQR 47-61), as compared to the median age in breast conserving surgery group (50 years, IQR 44-57), p<0.001; also a reflection of statin users being older on average.

**Table 1:** Patient and tumour characteristics by statin use.

Characteristic		Statin users, No. (%)	Nonusers, No. (%)	p-value
**All**		1353 (100)	6505 (100)	
**Age at diagnosis, years**	≤39	8 (0.6)	790 (12.1)	< 0.001
	40-49	209 (15.4)	2204 (33.9)	
	50-59	474 (35.0)	2162 (33.2)	
	60-69	485 (35.8)	998 (15.3)	
	70-79	155 (11.5)	287 (4.4)	
	≥80	22 (1.6)	64 (1.0)	
**Menopausal status**	Premenopausal	234 (17.3)	2816 (43.3)	< 0.001
	Postmenopausal	898 (66.4)	2500 (38.4)	
	Unknown	221 (16.3)	1189 (18.3)	
**Stage**	DCIS	197 (14.6)	920 (14.1)	0.975
	I	415 (30.7)	2006 (30.8)	
	II	486 (35.9)	2331 (35.8)	
	III	255 (18.8)	1248 (19.2)	
**Histology**	DCIS	197 (14.5)	921 (14.1)	0.347
	Infiltrative ductal	1044 (77.2)	4955 (76.2)	
	Infiltrative lobular	57 (4.2)	291 (4.5)	
	Other	55 (4.1)	338 (5.2)	
**Grade**	I	203 (15.0)	962 (14.8)	0.937
	II	520 (38.4)	2442 (37.5)	
	III	596 (44.1)	2866 (44.1)	
	Unknown	34 (2.5)	235 (3.6)	
**ER status**	ER positive	981 (72.5)	4713 (72.5)	0.954
	ER negative	306 (22.6)	1464 (22.5)	
	Unknown	66 (4.9)	328 (5.0)	
**HER2 status (Stage I-III only)**	HER2 positive	281 (24.3)	1406 (25.2)	0.518
	HER2 negative	805 (69.6)	3835 (68.7)	
	Equivocal/Unknown	70 (6.1)	344 (6.2)	
**Molecular subtype(Stage I-III only)**	HR+/HER2-	698 (60.4)	3276 (58.7)	0.074
	HR+/HER2+	153 (13.2)	873 (15.6)	
	HR-/HER2+	127 (11.0)	531 (9.5)	
	HR-/HER2-	106 (9.2)	559 (10.0)	
	Unknown	72 (6.2)	346 (6.2)	
**Type of surgery**	Breast conservation surgery	423 (31.3)	2567 (39.5)	< 0.001
	Mastectomy	888 (65.6)	3618 (55.6)	
	Others	3 (0.2)	16 (0.2)	
	Unknown	39 (2.9)	304 (4.7)	
**Chemotherapy**	Yes	684 (50.6)	3270 (50.3)	0.894
	No	500 (37.0)	2411 (37.1)	
	Unknown	169 (12.5)	824 (12.7)	
**Hormone therapy**	Yes	910 (67.3)	4447 (68.4)	0.423
	No	443 (32.7)	2057 (31.6)	
	Unknown	0 (0)	1 (0.02)	
**Radiotherapy**	Yes	736 (54.4)	4001 (61.5)	< 0.001
	No	419 (31.0)	1606 (24.7)	
	Unknown	198 (14.6)	898 (13.8)	
**Comorbidities at diagnosis**	Aspirin use, pre and post	200 (14.8)	114 (1.8)	< 0.001
	Diabetes mellitus	181 (13.4)	391 (6.0)	< 0.001
	Hypertension	165 (12.2)	402 (6.2)	< 0.001
	Cardiac disease	4 (0.3)	9 (0.1)	0.258
	Peripheral vascular disease	0 (0)	0 (0)	NA
	CNS disease	8 (0.6)	8 (0.1)	0.003
	Hyperlipidemia	112 (8.3)	186 (2.9)	< 0.001
	Renal disease	165 (12.2)	406 (6.2)	< 0.001

DCIS, Ductal Carcinoma in Situ; ER, Estrogen Receptor; HER2, Human Epidermal Growth Factor Receptor 2; HR, Hormone Receptor (i.e. Estrogen and/or progesterone receptors); CNS, central nervous system; NA, not applicable.

Patients were followed up for a median of 8.74 years, with a 95% confidence interval of 8.59 to 8.88 years. **Table 2** shows the unadjusted and adjusted hazard ratios for breast cancer recurrence by statin use in different subsets of patients.

**Table 2:** Unadjusted and adjusted hazard ratios for breast cancer recurrence by statin use in different subsets of patients.

	No. of events (person-years)	Unadjusted hazard ratio (95% CI)	p-value	Adjusted hazard ratio (95% CI)^~	p-value~
**All patients**					
Nonusers	1158 (43845)	Reference		Reference	
Statin users (1-5 years)	77 (3066)	0.90 (0.71 – 1.13)	0.361	0.98 (0.77 – 1.26)	0.898
Statin users (6-10 years)	30 (2771)	0.54 [0.61 (0.39 – 0.95)]	[0.028]	0.48 [0.49 (0.30 – 0.80)]	[0.005]
* Overall p-value for statin users*			*0.002*		*0.002*
**All ER +**					
Nonusers	836 (31570)	Reference		Reference	
Statin users	67 (4216)	0.63 (0.49 – 0.81)	<0.001	0.59 (0.45 – 0.78)	<0.001
**All ER -**					
Nonusers	289 (9624)	Reference		Reference	
Statin users (1-5 years)	31 (668)	1.54 (1.06 – 2.25)	0.024	2.03 (1.37 – 3.02)	<0.001
Statin users (6-10 years)	7 (637)	0.61 [0.40 (0.17 – 0.95)]	[0.038]	0.65 [0.32 (0.12 – 0.86)]	[0.025]
* Overall p-value for statin users*			*0.044*		*0.003*
**DCIS**					
Nonusers	96 (6815)	Reference		Reference	
Statin users	14 (818)	1.30 (0.73 – 2.29)	0.371	1.24 (0.64 – 2.40)	0.525
**Stage I-III**					
Nonusers	1062 (37030)	Reference		Reference	
Statin users (1-5 years)	70 (2685)	0.86 (0.67 – 1.09)	0.215	0.94 (0.72 – 1.21)	0.620
Statin users (6-10 years)	23 (2334)	0.46 [0.53 (0.33 – 0.88)]	[0.013]	0.48 [0.51 (0.31 – 0.84)]	[0.009]
* Overall p-value for statin users*			*<0.001*		*0.002*
**Stage I-III, ER+**					
Nonusers	773 (27135)	Reference		Reference	
Statin users	59 (3706)	0.59 (0.45 – 0.76)	<0.001	0.57 (0.43 – 0.76)	<0.001
**Stage I-III, ER-**					
Nonusers	269 (8669)	Reference		Reference	
Statin users (1-5 years)	29 (592)	1.56 (1.06 – 2.30)	0.026	1.95 (1.30 – 2.93)	0.001
Statin users (6-10 years)	5 (556)	0.51 [0.33 (0.12 – 0.89)]	[0.029]	0.66 [0.34 (0.12 – 0.93)]	[0.036]
* Overall p-value for statin users*			*0.031*		*0.007*
**Stage I-III, ER+HER2-**					
Nonusers	600 (21151)	Reference		Reference	
Statin users	48 (2944)	0.60 (0.44 – 0.80)	0.001	0.59 (0.43 – 0.80)	0.001
**Stage I-III, ER+HER2+**					
Nonusers	143 (4979)	Reference		Reference	
Statin users	10 (637)	0.60 (0.31 – 1.14)	0.119	0.46 (0.22 – 0.95)^@^	0.037
**Stage I-III, ER-HER2+**					
Nonusers	98 (4020)	Reference		Reference	
Statin users (1-5 years)	14 (320)	1.68 (0.95 – 2.98)	0.073	2.00 (1.10 – 3.64)	0.023
Statin users (6-10 years)	1 (305)	0.30 [0.18 (0.02 – 1.48)]	[0.111]	0.37 [0.13 (0.02 – 1.12)]	[0.064]
* Overall p-value for statin users*			*0.094*		*0.031*
**Stage I-III, TNBC**					
Nonusers	127 (3445)	Reference		Reference	
Statin users	18 (398)	1.51 (0.92 – 2.48)	0.106	1.83 (1.07 – 3.13)	0.026

~Hazard ratios, 95% CIs and p values in square brackets are for the time dependent term which allowed the hazard ratio for statin use to change after 5 years. The hazard ratio for 6-10 years was obtained by multiplying the hazard ratio for 1-5 years by this term. ^@^Statin use was significantly non proportional in this model. However, the time dependent term could not be fitted as there were no events amongst statin users after 5 years.

ER, Estrogen Receptor; DCIS, Ductal Carcinoma in Situ; HER2, Human Epidermal Growth Factor Receptor 2; TNBC, Triple negative breast cancer. ^Where possible, models were adjusted for age at diagnosis, TNM stage, histological grade, ER status, HER2 status, aspirin use (yes or no; time-varying, lagged by 1 year) and the presence of the abovestated co-morbidities at diagnosis. Variables which were significantly non-proportional were modeled using a time-dependent coefficient which allowed the hazard ratio to change after 5 years.

Comparing the statin user and non-user groups, when adjusted for age, and stage, statin users had similar risk of recurrence compared to non-statin users in the first 5 years of follow up (HR 0.98, 95%CI, 0.77-1.26). However, after 5 years, the risk of recurrence decreased significantly amongst statin users, with the hazard ratio estimated to be 0.48 after 5 years, p=0.002. This beneficial trend was seen in statin users with invasive cancers, beyond 6 years (HR 0.48, p= 0.002), but not so for statin users who had DCIS (HR 1.24, p=0.525).

ER positive cancers (DCIS inclusive) had a lower risk of recurrence in statin users (HR 0.59, 95%CI 0.45-0.78, p<0.001). On the contrary, there appeared to be an increased risk of recurrence in statin users who had ER negative cancers in the first 6 years post surgery (HR 2.03, p<0.001), but this risk of recurrence in statin users decreased significantly beyond the first 6 years (HR =0.65, p=0.003). It is uncertain if this increased risk was due to statin use itself, but it was more likely associated with the statin user not being accounted for in the model.

The benefit of statins in ER positive cancers was different between the *in-situ* and invasive groups. In the invasive cancer group, statin use was associated with a reduced risk of recurrence in the ER positive cancers (HR 0.57, 95%CI 0.43-0.76, p<0.001), and this is observed in both ER+ HER2-invasive cancers (HR 0.59, 95%CI 0.43-0.80,p=0.001) and ER+ HER2+ invasive cancers (HR 0.46, 95%CI 0.22-0.95, p=0.037). There appeared to be no statistically significant influence of statin in both the ER+ and ER- DCIS cancers.

Although there was no risk reduction benefit of statins on the ER- and HER2+ invasive cancers in the first 6 years, the risk of recurrence in both these ER- and HER2+ groups decreased significantly beyond the sixth year of diagnosis (HR 0.66, p=0.007 and HR 0.10, p=0.002 respectively).There was also an overall risk reduction benefit of statin in HER2- breast cancers, HR 0.74, p=0.026.

For disease-specific survival (**Table 3**), there was a benefit for statin users who were ER positive (adjusted SHR 0.71, 95%CI 0.53-0.96,p=.0.027), especially the ER+ invasive cancers (adjusted SHR 0.72, 95%CI 0.53-0.97, p=0.028) and ER+ HER2- invasive cancers (adjusted SHR 0.69, 95% CI 0.49-0.97, p=0.035). There was a suggestion of an improved overall survival in statin users who had ER+ cancers (HR=0.81) and ER+ invasive cancers (HR 0.79), but there was no statistically significant difference, albeit close (p=0.087 and p=0.061 respectively) There was no significant benefit in improved overall survival (**Table 4**) in all subtypes of breast cancer patients.

**Table 3:** Unadjusted and adjusted subhazard ratios for disease-specific survival by statin use in different subsets of patients.

	No. of events (person-years)	Unadjusted subhazard ratio (95% CI)	p-value	Adjusted subhazard ratio (95% CI)^	p-value
**All patients**					
Nonusers	623 (46768)	Reference		Reference	
Statin users	100 (6244)	1.17 (0.94 – 1.45)	0.151	1.05 (0.83 – 1.33)	0.702
**All ER positive**					
Nonusers	422 (33737)	Reference		Reference	
Statin users	56 (4477)	0.91 (0.68 – 1.20)	0.498	0.71 (0.53 – 0.96)	0.027
**All ER negative**					
Nonusers	181 (10259)	Reference		Reference	
Statin users	41 (1445)	1.84 (1.31 – 2.59)	<0.001	2.18 (1.51 – 3.15)	<0.001
**DCIS**					
Nonusers	9 (7173)	Reference		Reference	
Statin users	1 (889)	0.72 (0.08 –6.05)	0.759	Not estimable (no events)	
**Stage I-III**					
Nonusers	614 (39596)	Reference		Reference	
Statin users	99 (5355)	1.16 (0.93 – 1.44)	0.180	1.05 (0.83 – 1.34)	0.675
**Stage I-III, ER+**					
Nonusers	415 (29072)	Reference		Reference	
Statin users	56 (3927)	0.91 (0.68 – 1.21)	0.508	0.72 (0.53 – 0.97)	0.028
**Stage I-III, ER -**					
Nonusers	180 (9241)	Reference		Reference	
Statin users	41 (1264)	1.91 (1.36 – 2.69)	<0.001	2.18 (1.51 – 3.15)	<0.001
**Stage I-III, HER2 +**					
Nonusers	144 (9604)	Reference		Reference	
Statin users	31 (1351)	1.55 (1.04 – 2.30)	0.032	1.34 (0.86 – 2.08)	0.199
**Stage I-III, HER2 -**					
Nonusers	431 (27358)	Reference		Reference	
Statin users	62 (3662)	1.03 (0.79 – 1.35)	0.819	0.97 (0.73 – 1.30)	0.861
**Stage I-III, ER+HER2-**					
Nonusers	320 (22650)	Reference		Reference	
Statin users	40 (3129)	0.82 (0.59 – 1.14)	0.233	0.69 (0.49 – 0.97)	0.035
**Stage I-III, ER+HER2+**					
Nonusers	80 (5323)	Reference		Reference	
Statin users	12 (660)	1.12 (0.60 – 2.08)	0.719	0.80 (0.41 – 1.56)	0.503
**Stage I-III,ER-HER2+**					
Nonusers	64 (4259)	Reference		Reference	
Statin users	19 (683)	2.13 (1.26 – 3.58)	0.005	2.01 (1.11 – 3.64)	0.022
**Stage I-III, TNBC**					
Nonusers	87 (3654)	Reference		Reference	
Statin users	21 (453)	2.22 (1.40 – 3.54)	0.001	2.43 (1.48 – 4.00)	<0.001

ER, Estrogen Receptor; DCIS, Ductal Carcinoma in Situ; HER2, Human Epidermal Growth Factor Receptor 2; TNBC, Triple negative Breast Cancer. ^Where possible, models were adjusted for age at diagnosis, TNM stage, histological grade, ER status, HER2 status, aspirin use (yes or no; time-varying, lagged by 1 year) and the presence of the abovestated co-morbidities at diagnosis. Variables which were significantly non-proportional were modeled using a time-dependent coefficient which allowed the hazard ratio to change after 5 years.

**Table 4:** Unadjusted and adjusted hazard ratios for overall survival by statin use in different subsets of patients.

	No. of events (person-years)	Unadjusted hazard ratio (95% CI)	p-value	Adjusted hazard ratio (95% CI)^	p-value
**All patients**					
Nonusers	834 (46884)	Reference		Reference	
Statin users	172 (6254)	1.50 (1.27 – 1.77)	<0.001	1.03 (0.84 – 1.25)	0.801
**All ER +**					
Nonusers	575 (33824)	Reference		Reference	
Statin users	112 (4487)	1.34 (1.10 – 1.65)	0.005	0.81 (0.64 – 1.03)	0.087
**All ER -**					
Nonusers	228 (10286)	Reference		Reference	
Statin users	52 (1445)	1.80 (1.32 – 2.44)	<0.001	1.71 (1.22 – 2.40)	0.002
**DCIS**					
Nonusers	32 (7177)	Reference		Reference	
Statin users	11 (893)	2.52 (1.25 – 5.08)	0.009	1.21 (0.51 – 2.91)	0.665
**DCIS, ER+**					
Nonusers	20 (4669)	Reference		Reference	
Statin users	6 (553)	2.16 (0.85 – 5.50)	0.107	1.13 (0.40 – 3.23)	0.819
**DCIS, ER-**					
Nonusers	6 (1018)	Reference		Reference	
Statin users	3 (182)	2.90 (0.71 – 11.90)	0.139	1.81 (0.41 – 8.06)	0.438
**Stage I-III**					
Nonusers	802 (39707)	Reference		Reference	
Statin users	161 (5362)	1.44 (1.21 – 1.71)	<0.001	1.01 (0.83 – 1.23)	0.918
**Stage I-III, ER+**					
Nonusers	555 (29155)	Reference		Reference	
Statin users	106 (3933)	1.30 (1.06 – 1.61)	0.014	0.79 (0.62 – 1.01)	0.061
**Stage I-III, ER-**					
Nonusers	222 (9268)	Reference		Reference	
Statin users	49 (1264)	1.79 (1.31 – 2.44)	<0.001	1.70 (1.21 – 2.40)	0.002
**Stage I-III, HER2+**					
Nonusers	186 (9638)	Reference		Reference	
Statin users	39 (1353)	1.48 (1.04 – 2.09)	0.029	1.10 (0.75 – 1.63)	0.622
**Stage I-III, HER2-**					
Nonusers	561 (27433)	Reference		Reference	
Statin users	108 (3667)	1.38 (1.12 – 1.70)	0.002	1.00 (0.79 – 1.25)	0.968
**Stage I-III, ER+HER-**					
Nonusers	426 (22706)	Reference		Reference	
Statin users	81 (3134)	1.26 (0.99 – 1.60)	0.058	0.83 (0.63 – 1.08)	0.163
**Stage I-III, ER+HER2+**					
Nonusers	105 (5349)	Reference		Reference	
Statin users	16 (662)	1.14 (0.67 – 1.93)	0.636	0.70 (0.38 – 1.28)	0.249
**Stage I-III, ER-HER2+**					
Nonusers	81 (4268)	Reference		Reference	
Statin users	23 (683)	1.94 (1.21 – 3.10)	0.006	1.70 (1.01 – 2.88)	0.048
**Stage I-III, TNBC**					
Nonusers	107 (3672)	Reference		Reference	
Statin users	25 (453)	2.06 (1.32 – 3.19)	0.001	1.94 (1.20 – 3.16)	0.007

ER, Estrogen Receptor; DCIS, Ductal Carcinoma in Situ; HER2, Human Epidermal Growth Factor Receptor 2; TNBC, Triple Negative Breast Cancer. ^Where possible, models were adjusted for age at diagnosis, TNM stage, histological grade, ER status, HER2 status, aspirin use (yes or no; time-varying, lagged by 1 year) and the presence of the abovestated co-morbidities at diagnosis. Variables which were significantly non-proportional were modeled using a time-dependent coefficient which allowed the hazard ratio to change after 5 years.

## Discussion

To the best of our knowledge, this is the first retrospective study of the impact of statins on the risk of breast cancer recurrence in an Asian population. The results from this study demonstrate that statin use after the diagnosis of breast cancer can potentially reduce the risk of breast cancer recurrence. This is observed in patients who had invasive cancers which either expressed the estrogen receptor (ER) and/or who are HER2 negative. This is similar to previous studies done in Europe and in the US, that showed the beneficial effect of statins in reducing the risk of recurrence in Stage I-III invasive breast cancers (14, 15, 31–35), and the beneficial effect of statins in reducing the risk of recurrence in ER positive patients (13, 15, 35).

There are a few possibilities to explain this interesting phenomenon. One theory is the direct reduction of estrogen production, through the statin induced reduction of cholesterol (precursor of estrogen hormones). Although lipid concentrations (which are also decreased by statins) do not seem to have an impact on breast cancer risk overall (36), cholesterol levels may still modulate the action of estrogen receptors (37).

Another possibility is the synergism between statins and the adjuvant hormonal therapy (tamoxifen or aromatase inhibitor) which is also administered to ER+ breast cancer patients in the first 5 years post surgery (35). This is assuming patients with ER positive cancers (which are similar in proportion in both statin and non-statin groups) are compliant to adjuvant hormonal therapy as they are with their statin use. There are *in vitro* studies that demonstrate how statins attenuate the growth of ER+ breast cancer cell lines (MCF-7) (38–43), as well as the synergistic effect of statins with tamoxifen (44) or exemestane (an aromatase inhibitor) (45) to induce cell apoptosis, through the downregulation of the expression of suvivin proteins (43, 44).

Another theory is the direct impact of statins on the mevalonate pathway, which produces cholesterol, steroid hormones, and non-steroid isoprenoids, which are necessary for cell survival (46). HMGCR (3-hydroxy-3-methylglutaryl–coenzyme A reductase) is differentially expressed among breast cancers, with higher expression in the tumour cells (*vs* normal epithelial cells) (47), but the correlation with its expression and breast cancer prognosis and survival is controversial. One study (48) showed that HMGCR moderate/strong expression was associated with prognostically adverse tumour characteristics (higher histological grade, high Ki67, and ER negativity) and that neither HMGCR expression nor statin use was associated with breast cancer mortality (48). This was also seen in a Korean cohort study (49) which showed that a higher tumour expression of HMGCR was associated with poor disease free survival, particularly in the TNBC group. Yet, some studies showed that a stronger expression of HMGCR was associated with a less aggressive tumour profile (low histological grade, a small tumour size, oestrogen receptor (ER) positivity, and low proliferation) (50, 51), and HMGCR-positive cancers had longer recurrence-free survival, which was more pronounced in patients with ER-positive tumours (48, 51).

Interestingly, we also observed that statins appear to reduce the risk of recurrence in all patients with invasive breast cancer beyond the sixth year of diagnosis (HR 0.48, overall p=0.002). This is also observed in those with ER- (HR 0.66, overall p=0.007) and HER2+ (HR 0.10, overall p=0.002) invasive breast cancers. This is also supported by *in vitro* studies, where statins can also attenuate the growth of triple negative breast cell lines (MDA-MB231) (38–40, 52), and reduce breast cancer cell migration (42, 53, 54). These suggest more complex pathway(s) in which statins contribute to cancer risk reduction, beyond their direct impact on the ER receptor and its pathway.

A possible explanation for this long term benefit of statins is that the triple negative (i.e. ER- PR-HER2-) breast cancers are generally more aggressive, have fewer available adjuvant systemic therapies, and have a higher risk of cancer recurrence within the first 5 years. This is opposed to ER positive cancers which tend to have a better prognosis, but longer periods of dormancy (55). Therefore, the beneficial effect of statins in reducing cancer recurrence in the longer-term may be attributed to their role in cancer dormancy. Tumour dormancy is a clinical phenomenon in which disseminated tumour cells remain occult, asymptomatic, and undetectable over a prolonged period of time. Tumour dormancy contributes to local recurrence or distal metastasis, up to years or decades after treatment (56). This may also explain why the protective role of statins in our DCIS population is not appreciated. DCIS refers to the proliferation of neoplastic epithelial cells within the tubulolobular system of the breast. Although DCIS shares morphological features of invasive breast carcinoma, they are confined by the myoepithelial cells and basement membrane of the ducts, without invasion of the stroma, lymphatic or blood vessels. DCIS, by definition is pre-invasive (Stage 0), and has a much lower risk of recurrence than invasive cancer.

This is further supported by the popular theory of the anti-inflammatory properties of statins, particularly on cytokines, in the tumour microenvironment. Statins have also been demonstrated *in vitro* to decrease interleukin-6 (IL6) production in breast cancer cell lines (57–60) and non-breast cancer cell lines (61–63). Lipophilic statins can also suppress the common cluster of genes (Hippo, Notch and Wnt pathways) governing the epithelial mesenchymal transition (EMT) process (64) - an important step in tumour dormancy, recurrence and metastasis. Other proposed anti-carcinogenic effects of statins include apoptosis (65–67) and inhibition of inflammation (66, 68, 69), proliferation, migration (39, 70, 71) and angiogenesis (72).

Majority of studies support an improvement in disease-specific survival (6, 16, 19, 24, 33, 73), but were without tumour subtype analysis. The findings in this study demonstrated a disease-specific survival benefit for statin users who were ER positive (HR 0.69, p=.0.023).This is appreciated in the ER+ invasive cancers (HR 0.70, p=0.024) and ER+ HER2- invasive cancers (HR 0.68, p=0.036), and is a reflection of the risk reduction benefit of statins on cancer recurrence in these ER+ invasive cancers. However, the benefit of statins reducing disease-specific survival in all breast cancer patients was not appreciated in this study, which may be in part due to small event numbers or an inadequately long enough follow-up (i.e.>10 years). There was a suggestion of an improved overall survival in statin users who had ER+ cancers (HR=0.81) and ER+ invasive cancers (HR 0.79), but there was no statistically significant difference, albeit close (p=0.087 and p=0.061 respectively). It is possible that the true benefit on overall survival may be better appreciated with a longer followup (i.e. > 10 years).

Similar to other studies (23, 33), there was no significant benefit in improved overall survival (**Table 4**) in all subtypes of breast cancer patients. This may be in part due to close surveillance and screening of breast cancer survivors, in picking up recurrence(s), if any, early, and the improved modality of systemic treatment. Also, statin users are also more likely to be older and have more comorbidities, which in themselves pose a risk of earlier mortality. Hence, after adjusting for these variables, there are no significant differences for overall and disease-specific survival between statin and non-statin users.

In this study, we chose to focus only on the post-diagnostic statin users, which is similar to most studies (14, 15, 22, 31–33, 74). This helps remove the confounders of statin use duration, and the bias that statin use in itself may reduce breast cancer risk (12, 31), and may have more favorable tumor characteristics and hence improved outcomes compared with statin nonusers (13).

The main limitation of this study is that it is a retrospective study in a single healthcare cluster. Nonetheless, SingHealth is the largest public healthcare cluster in Singapore. A large majority of our patients have long term follow up, with a median of 8.674 years, with a 95% confidence interval of 8.59 to 8.88 years. As such, the possible biases that might have been introduced due to a loss of follow-up is small. The probability of patients who were lost to follow up and had a greater proportion of statin users and recurrence than the rest of the patients who completed the study is also low.

Another assumption for this study is that the first electronic prescription of statins is the patients’ index prescription and hence the start date of statins. As the largest public healthcare cluster, we have a good electronic prescription record that also links up with the primary care physicians clinics within the cluster. All patients who undergo oncological surgery in hospital will have at least one record of their prescriptions on the day of their surgery when they are admitted, and hence any patients who are already on existing statin prescription will be detected and excluded from this study. Therefore, it is reasonable to assume that any prescription after the date of surgery will accurately represent the start of statins post surgery. We acknowledge that there may be a group of patients who are prescribed statins by their private practitioners or through another healthcare group post-diagnosis and this group of patients may contribute to a misclassification bias. This group is estimated to be small as the majority of patients tend to be followed up with the SingHealth primary health care, and/or have their usual prescriptions topped up during their oncologist visits. More importantly, patients who had a recurrence, and hence included in this study, would have been worked up in the treating institution at the point of recurrence, and have their electronic prescriptions reviewed, hence minimizing the chances of the misclassification bias. In addition, we have no reason to believe that the proportion of statin users erroneously classified would be too dissimilar in both outcome groups—those who recurred and those who remained free of disease. In that sense, misclassification errors should be non-differential and generally this tend to bias the study toward null hypothesis, not against it.

Another assumption of this study is the compliance of statins once the patient is started on it. We can only assume that a prescription represents compliance to consuming the medication, and that the patient complies life-long with this cardioprotective cholesterol-lowering statin. We can infer this through top-up prescriptions, but acknowledge that patients may also get their top-up prescription through other healthcare providers. There is also the assumption that patients who are more compliant with their medications, especially chronic medications, are more likely to be healthier and adherent to other prescribed drugs, such as the adjuvant hormonal therapeutic agents with tamoxifen or aromatase inhibitors. If this were the case, then a selection bias might have also played a role in this study.

Also, the vast majority of the patients in this study were prescribed a lipophilic statin (96.3%), with simvastatin and atorvastatin being the most common statins used. With this skewed preference for lipophilic statins in this population, a comparison between hydrophilic vs lipophilic statins and their impact on breast cancer recurrence was not feasible.

Another potential limitation of our study is that we could not adjust for body mass index and other lifestyle variables, such as smoking and alcohol use because the data collection for this was incomplete. A previously published study in an overlapping population showed that body mass index was associated only with distant breast cancer recurrences (75). Nonetheless, this paper has its strengths. It has a large number of patients (n=7858) with long follow-up median of 8.59 years. We were also able to analyze and compare the different subtypes of breast cancer - comparing the DCIS *vs* invasive breast cancers, as well as the different immunohistochemical subtypes of invasive breast cancer. Despite possible genetic differences in Asian breast cancer, pharmacokinetic differences in statin metabolism and lifestyle difference, we have demonstrated that, similar to previous international studies, statin use can help reduce a risk of breast cancer recurrence. This is especially beneficial in patients who have ER+ invasive breast cancer. We have demonstrated that statin use can potentially reduce breast cancer recurrence in all subtypes of breast cancer in the long term (> 6 years post diagnosis). The underlying mechanisms of its beneficial action is still yet to be fully elucidated.

We look forward to prospective clinical trials (such as NCT04601116, NCT03971019, NCT04705909, NCT00914017 and NCT02958852, and not exhaustive), to enlighten us on the true effects of statins in preventing breast cancer risk and recurrence; and if it outweighs the small, but known side effects, such as statin-associated muscle symptoms (SAMS) (76, 77), effect on glucose homeostasis and hepatic toxicity (77). Further *in vitro* and translation studies are also needed to understand the pathways in which statins exerts its anti-carcinogenic effects.

## Conclusion

This is the first retrospective study in Asian population to study the effect of statin use and breast cancer recurrence. Despite possible genetic differences in Asian breast cancer, pharmacokinetic differences in statin metabolism and lifestyle difference, we have demonstrated that, similar to previous international studies, statin use can help reduce a risk of breast cancer recurrence. This is especially beneficial in patients who have ER+ and/or HER2- invasive breast cancer. We have also demonstrated that statin use potentially reduce breast cancer recurrence in all subtypes of breast cancer in the long term (> 6 years post diagnosis). This risk reducing effects of statins on breast cancer recurrence, coupled with their cardioprotective effect, demonstrate the underlying complexity in cancer pathways and metabolism, and may open up new potential anti-cancer targets for future therapeutics.

## Data Availability Statement

The raw data supporting the conclusions of this article will be made available by the authors, without undue reservation.

## Ethics Statement

The studies involving human participants were reviewed and approved by SingHealth institutional ethics board (CIRB 2019/2419). Written informed consent for participation was not required for this study in accordance with the national legislation and the institutional requirements.

## Author Contributions

YS contributed to the conception and design of the study, and the writing of the manuscript. NP contributed to the retrieval of data and organisation of the database and YS, CL, and NP contributed to the organization of the database. CL performed the statistical analysis and contributed to the design of the study. All authors contributed to manuscript revision, read and approved the submitted version.

## Funding

This project was supported by the National Cancer Centre Singapore (NCCS) Cancer fund.

## Conflict of Interest

The authors declare that the research was conducted in the absence of any commercial or financial relationships that could be construed as a potential conflict of interest.

## Publisher’s Note

All claims expressed in this article are solely those of the authors and do not necessarily represent those of their affiliated organizations, or those of the publisher, the editors and the reviewers. Any product that may be evaluated in this article, or claim that may be made by its manufacturer, is not guaranteed or endorsed by the publisher.

## Abbreviations

DCIS, Ductal Carcinoma in Situ; ER, Estrogen Receptor; HER2, Human Epidermal Growth Factor Receptor; HMG-CoA Reductase, HMGCR 3-hydroxy-3-methylglutaryl–coenzyme A reductase; HR, Hazard Ratio; SHR, Subhazard ratio; TNBC, Triple Negative Breast Cancer.
